# Evolutionary chemical binding similarity approach integrated with 3D-QSAR method for effective virtual screening

**DOI:** 10.1186/s12859-020-03643-x

**Published:** 2020-07-14

**Authors:** Prasannavenkatesh Durai, Young-Joon Ko, Cheol-Ho Pan, Keunwan Park

**Affiliations:** 1 Natural Product Informatics Research Center, KIST Gangneung Institute of Natural Products, Gangneung, 25451 Republic of Korea; 2 Department of Bioinformatics and Life Science, Soongsil University, Seoul, 06978 Republic of Korea

**Keywords:** Evolutionary chemical binding similarity, Virtual screening, 3D-QSAR, Machine learning, Ligand similarity, Pharmacophore, Molecular docking

## Abstract

**Background:**

Despite continued efforts using chemical similarity methods in virtual screening, currently developed approaches suffer from time-consuming multistep procedures and low success rates. We recently developed a machine learning-based chemical binding similarity model considering common structural features from molecules binding to the same, or evolutionarily related targets. The chemical binding similarity measures the resemblance of chemical compounds in terms of binding site similarity to better describe functional similarities that arise from target binding. In this study, we have shown how the chemical binding similarity could be used in virtual screening together with the conventional structure-based methods.

**Results:**

The chemical binding similarity, receptor-based pharmacophore, chemical structure similarity, and molecular docking methods were evaluated to identify an effective virtual screening procedure for desired target proteins. When we tested the chemical binding similarity method with test sets of 51 kinases, it outperformed the traditional structural similarity-based methods as well as structure-based methods, such as molecular docking and receptor-based pharmacophore modeling, in terms of finding active compounds. We further validated the results by performing virtual screening (using the chemical binding similarity and receptor-based pharmacophore methods) against a completely blind dataset for mitogen-activated protein kinase kinase 1 (MEK1), ephrin type-B receptor 4 (EPHB4) and wee1-like protein kinase (WEE1). The in vitro kinase binding assay confirmed that 6 out of 13 (46.2%) for MEK1 and 2 out of 12 (16.7%) for EPHB4 were newly identified only by the chemical binding similarity model.

**Conclusions:**

We report that the virtual screening results could further be improved by combining the chemical binding similarity model with 3D-QSAR pharmacophore and molecular docking models. Not only the new inhibitors are identified in this study, but also many of the identified molecules have low structural similarity scores against already reported inhibitors and that show the revelation of novel scaffolds.

## Background

The discovery of highly potent lead compounds is one of the most important steps in drug discovery. There has been a massive and continuous effort to develop an efficient lead discovery process because most identified hits are eventually dropped from the lead optimization procedure owing to low efficacy, poor bioavailability, or high toxicity [1, 2]. As an alternative to the tedious, expensive, and time-consuming experimental screening procedure, computational methods that can screen the vast amount of chemical compounds (virtual chemical library) have become an indispensable tool in the early stage of drug discovery [2]. Of these, chemical similarity calculation has been extensively used in drug discovery and proven effective in various applications such as lead discovery and target identification [3, 4].

Various virtual screening (VS) approaches have employed 2D and 3D molecular comparison methods that are based on fingerprints, substructures, and descriptors that enable simple, fast and computationally inexpensive analysis [4, 5]. Similarity Ensemble Approach [6] and SuperPred [7] are web based target prediction tools that use the 2D fingerprint similarity principle to compare the input molecules to available ligands (with target information) in the database. SwissTarget considers both 2D fingerprints and 3D electroshape vectors for molecular comparison to provide information on possible binding targets [8]. SpiDER [9] is another tool that compares the query molecule with self-organizing maps through pharmacophore features and predicts binding targets for a given molecule that may be an existing molecule or a designed scaffold. The SHAFTS approach in ChemMapper [10] suggests targets for chemicals based on both 3D pharmacophore mapping and shape overlay methods to compare molecules. Even though several tools are available for target prediction and drug identification, most of the methods are based on the structural similarity of chemical compounds that often fail to represent the functional biological activity derived from a specific combination of local spatial features [11]. Consideration of only structural similarities based on single target binding molecules may not be suitable for VS as the term activity cliffs are referred to compounds with high structural similarity but high activity differences [12].

The phenotypic similarity of chemicals is possibly calculated to represent functional activity similarity, irrespective of structural component similarity. For example, gene expression databases such as Connectivity Map [13] and the Library of Integrated Network-based Cellular Signatures [14] are used to identify chemical similarity. In phenotypic drug-drug similarity, two molecules are similar if they share similar gene expression, activities, or mechanisms for a query gene signature. The side-effect similarity of drugs is another approach to define whether two different drugs share the same or similar targets if they cause similar side-effects through drug-disease relationships [15, 16]. Although these methods directly encode biological activity in the similarity calculation, the phenotypic information of most chemical compounds is highly limited so it is difficult to use in a general VS task.

We recently developed a machine learning-based chemical similarity model referred to as a target-specific ensemble evolutionary chemical binding similarity (TS-ensECBS) model that was designed to measure the probability that chemical compounds bind to identical targets [17, 18]. The TS-ensECBS model encodes evolutionarily conserved key molecular features required for target-binding into the chemical similarity score, making it an optimal similarity method to screen novel candidate molecules to check for biological activity. Indeed, in our previous work [17], the TS-ensECBS model outperformed the traditional ligand-based similarity methods in terms of finding similar target-binding compounds.

In the present study, we compared the TS-ensECBS model with well-known ligand- and structure-based VS approaches to recommend a cohesive route for effective early stage lead identification. The chemical binding similarity method was tested on 51 kinases and compared to traditional structural similarity-based methods in terms of finding active compounds as well as structure-based methods such as molecular docking and receptor-based pharmacophore modeling. The chemical binding similarity was further validated by performing VS for kinases against a completely blind chemical dataset. Lastly, considering the distinct characteristics (prediction performance, running time, underlying principle, and requirement to run) of VS methods, we proposed an integrative screening approach adopting the TS-ensECBS model.

## Results

### Performance of virtual screening methods for the large-scale kinase test set

**Fig. 1:**
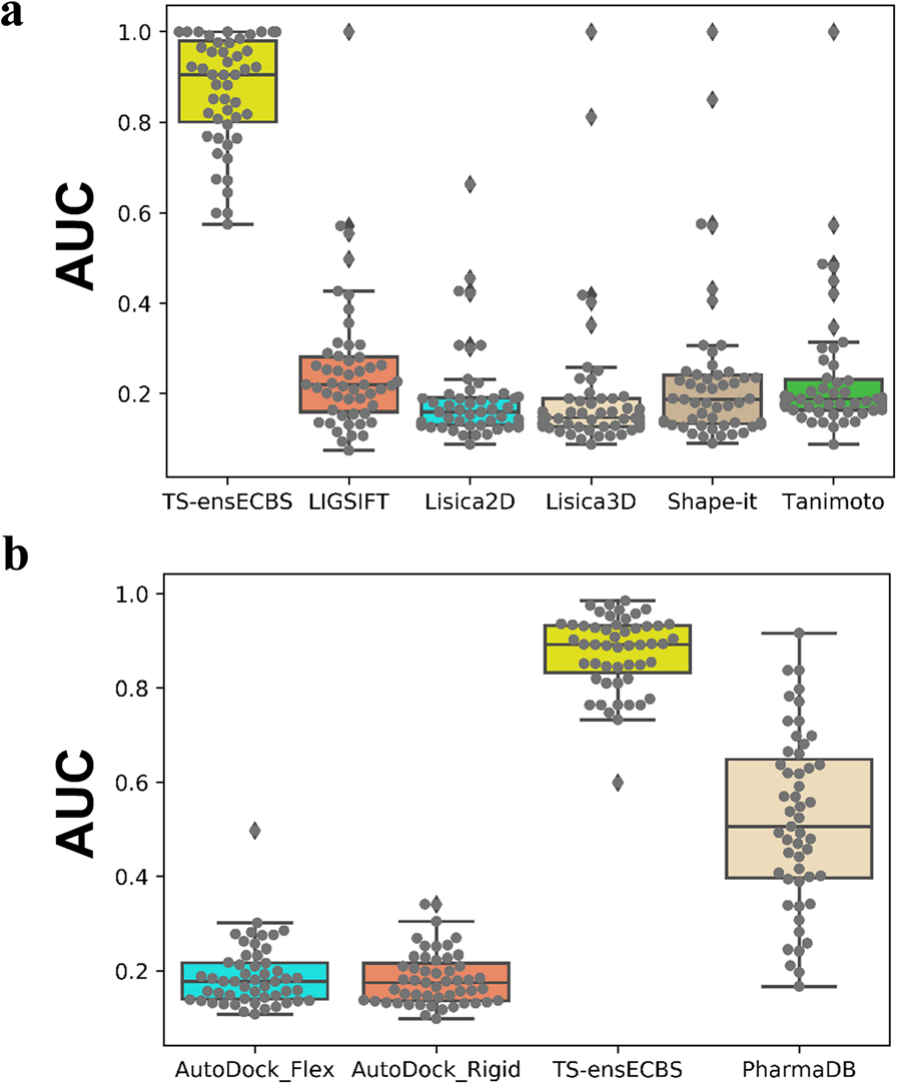
Validation of different virtual screening methods for the kinase test set. The AUC values in the precision-recall curve calculated for data of each kinase are shown for each method tested. All methods were validated on the 51 selected kinases using their known inhibitors along with unknown molecules chosen based on random selection. **a** The LIGSIFT, ligand similarity using clique algorithm (LiSiCA) 2D, LiSiCA 3D, shape-it, and Tanimoto ligand-based structural similarity methods were used for comparison with the TS-ensECBS method. **b** The pharmacophore model (PharmaDB) incorporated in Discovery studio 2018, AutoDock VINA with both rigid and flexible side chains, the structure-based methods employed for comparison with the TS-ensECBS method. Two different test sets were used for ligand-based and structure-based methods. AUC values were calculated for each kinase and their distributions are shown in Figs. 1 and SI Fig. 6. The TS-ensECBS model clearly outperformed the ligand-based 2D and 3D chemical similarity and structure-based methods, although the pharmacophore model performed reasonably. However, molecular docking performed poorly, regardless of the sidechain flexibility in the binding site. Therefore, we decided to test the TS-ensECBS and receptor-based pharmacophore models further for unseen chemical databases

The different VS methods (TS-ensECBS model, ligand-based shape similarity score, receptor-based pharmacophore model, and molecular docking with either flexible or rigid sidechains) were commonly applied to the kinase test set and their prediction accuracy was compared using area under the curve (AUC) values in a precision and recall (PR) curve as shown in Fig. 1. The TS-ensECBS model was a machine learning-based method trained using precompiled protein-ligand interaction data. In cases of structural similarity between chemicals, it was calculated through the molecular information obtained from chemical structures and easily applicable to VS using the similarity score to known active molecules. The chemical pairs were sorted by each chemical similarity method to compare the performance for finding common target-binding chemical pairs (Fig. 1a). The TS-ensECBS model showed higher performance than the structure similarity methods due to the encoded target-binding information in the training data.

In contrast, the receptor-based pharmacophore model was a structure-based method that extracted a set of critical steric and electrostatic features from a known protein-ligand complex structure. Molecular docking only required knowledge of the receptor structure without any prior binding site information and the calculated binding energy was used to score chemical compounds. For the TS-ensECBS model, chemical compounds were scored by assigning the maximum TS-ensECBS score to each test molecule. The result suggested that the TS-ensECBS model showed higher performance to prioritize the chemical compounds binding to a VS target (Fig. 1b).

The systematic comparison of these methods not only provided a performance estimation of the individual methods but also revealed how to combine them for improving VS procedures. The detailed steps about combination of effective VS procedure can be found in ‘Discussion’ part.

### Selection of kinases for the blind virtual screening test

For further performance validation, a small number of kinase targets were selected to find novel inhibitory molecules from completely unseen chemical databases. We focused on three kinases (mitogen-activated protein kinase kinase 1 (MEK1), ephrin type-B receptor 4 (EPHB4), and wee1-like protein kinase (WEE1)) that were selected by the following criteria based on the screening results of the test set shown in Fig. 1; a TS-ensECBS model PR AUC value greater than 0.8 (expecting high prediction accuracy), a number of known binding chemicals for the targets greater than 10 (ensuring sufficient training data), an output TS-ensECBS score for the positive chemicals higher than 0.8, and average score differences between positive and negative chemicals over 0.4 (expecting high discriminative power). These criteria were defined to select kinases with reliable, high predictive performance models likely to have high accuracy in the blind test. The PR AUC values of TS-ensECBS models were 0.93, 0.92, and 0.89 for MEK1, EPHB4, and WEE1, respectively. Similarly, the corresponding PR AUC values of the pharmacophore models were 0.68, 0.61, and 0.92, respectively. Hence, pharmaDB was used along with TS-ensECBS to further screen the unknown databases.

### Virtual screening for the three selected kinases

**Fig. 2:**
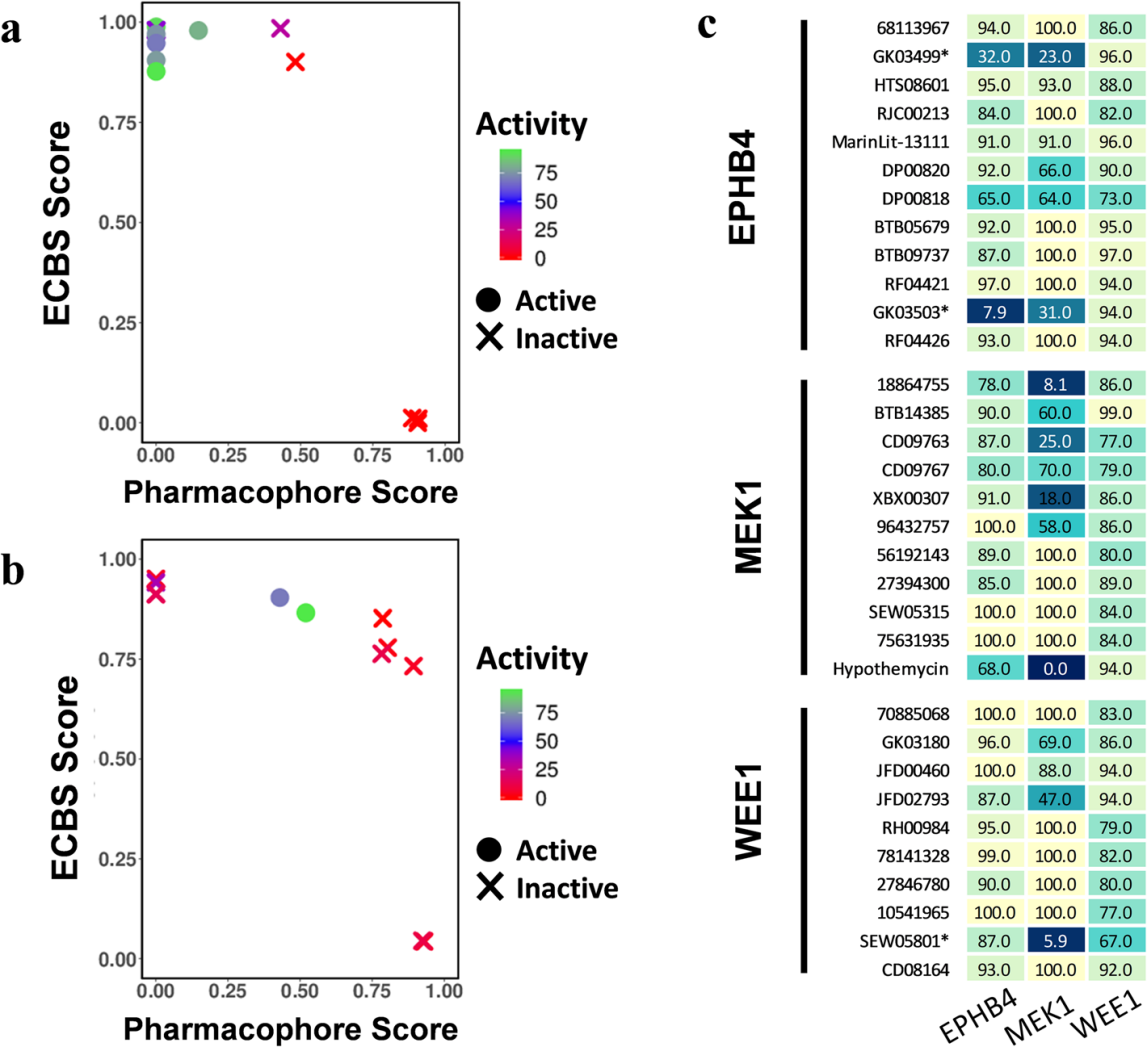
The TS-ensECBS and pharmacophore scores of the candidate molecules tested using an in vitro binding assay. The output score obtained from the TS-ensECBS model and PharmaDB screening for the molecules verified via the in vitro binding assay are shown. **a** MEK1 and **b** EPHB4. **c** all in vitro binding assay results (percentage of control (POC) values) are represented by a heat map where the lower POC value indicates a higher binding affinity. * indicates the molecules were also selected to assess MEK1 inhibitory activity. The activity values were derived from the POC value of the in vitro binding assay. [100 – POC] value is considered as the activity value shown. A lower POC value likely represents a higher binding affinity because the lowest percentage of the control in solid support means there was competition between a test molecule and an immobilized molecule for binding to the kinase

To find promising candidates in the chemical databases, we prioritized the chemical compounds by TS-ensECBS or receptor-based pharmacophore score. First, chemical compounds were only selected by TS-ensECBS score (cutoff 0.7), which resulted in 34, 53, and 238 for MEK1, EPHB4, and WEE1, respectively. Of these, the top 10 individual hits by TS-ensECBS and receptor-based pharmacophore score were shortlisted for each kinase target. Notably, only six molecules had a pharmacophore fit score above zero for MEK1. From those constricted hits, we excluded molecules that were out of stock, duplicates obtained as repetitive hits in more than one method, and those already reported as inhibitors for MEK1, EPHB4, and WEE1 kinases or closely associated kinases. To further compare PharmaDB and TS-ensECBS methods, the chemical databases were also later screened using only the PharmaDB receptor-based pharmacophore models. The PharmaDB models stored in PDB IDs 3v01, 4aw5, and 1x8b were used to select the candidate molecules using the pharmacophore fit score. The experimentally tested molecules had diverse score ranges for both TS-ensECBS and pharmacophore fit score (Fig. 2).

### In vitro kinase binding assay

**Fig. 3:**
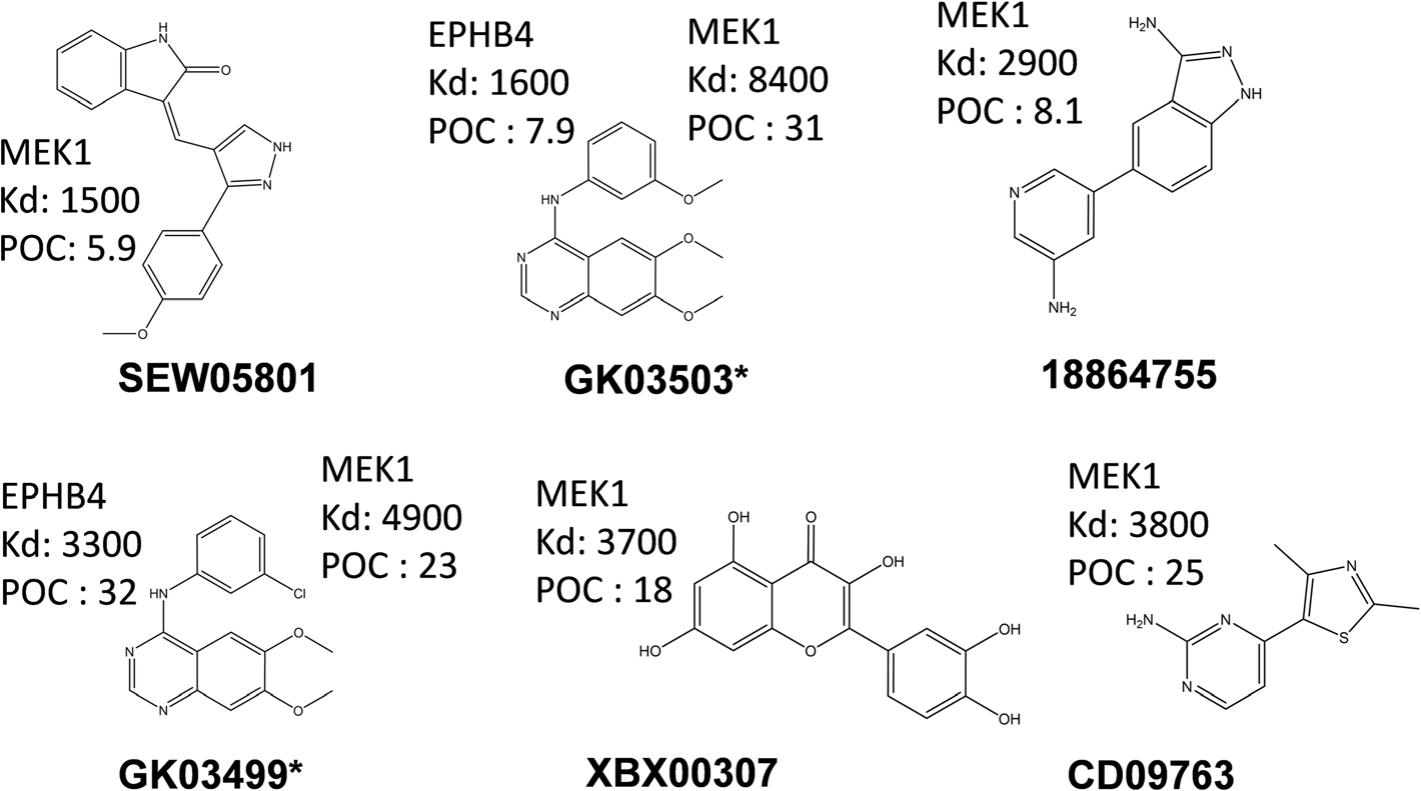
Two-dimensional structures of the experimentally validated lead compounds. Every lead molecule with its two-dimensional structure, name of its binding target, binding constant (Kd in nM) and percentage of control (POC) values are given. * represents dual inhibitors of MEK1 and EPHB4

An in vitro binding assay was used to test the final candidate molecules for binding specificity and binding affinity. As a positive control, hypothemycin, a predicted but known inhibitor of MEK1, was also included to perform the experimental assay. Out of 32 molecules tested through the in vitro binding assay with 10 μM concentration, SEW05801, 18864755, XBX00307, and CD09763 were successfully found to be potential inhibitors of MEK1 and GK03499 and GK03503 were found to be dual inhibitors for MEK1 and EPHB4 (Figs. 2 and 3). The percentage of a kinase in solid support (that confirms the competition between a test molecule and an immobilized molecule for binding to a kinase) varied between 5.9 and 32% (Fig. 2 and Table 1). For the eight active molecules (Fig. 3), we also calculated the dissociation constant (Kd) (SI Figs. 1 and 2) and the values were between 1500 nM and 8400 nM (Table 1). Contrastingly, no active molecules were found for WEE1 that can be seen along with kinase diversity in SI Fig. 3. Out of eight active molecules, only three had low to moderate pharmacophore scores from PharmaDB screening (0.15, 0.44, and 0.52; Table 1). Nineteen molecules had pharmacophore fit scores above zero but only three of them were active in the competitive binding assay. Hence, receptor-based pharmacophore models may not be suitable for final scoring in VS.

**Table 1:** Competitive binding assay hits. Compounds identified in this study. * represents dual inhibitors of MEK1 and EPHB4

Compound	Target	TS-ensECBS score	Pharmacophore fit score	Percent of control (POC)	Kd (nM)	Rigid docking (kcal/mol)	Flexible docking (kcal/mol)	Shape similarity
SEW05801	MEK1	0.87	0	5.9	1500	−8.4	−10.4	0.47
18864755	MEK1	0.98	0	8.1	2900	−9.7	−9.8	0.52
XBX00307	MEK1	0.97	0.15	18	3700	−8.6	−9.6	0.59
CD09763	MEK1	0.97	0	25	3800	−6.3	−7	0.43
GK03499*	MEK1	0.9	0	23	4900	−8.2	−9.8	0.76
GK03503*	MEK1	0.94	0	31	8400	−8.2	−9.8	0.85
GK03503*	EPHB4	0.86	0.52	7.9	1600	−8.2	−9	0.72
GK03499*	EPHB4	0.9	0.44	32	3300	−8.5	−9.5	0.67

In contrast, the TS-ensECBS model consistently showed high performance in selecting potential inhibitors for both MEK1 and EPHB4. All active molecules showed high TS-ensECBS scores (above 0.87), whereas candidate molecules selected only using pharmacophore fit scores were all inactive (Fig. 2). Molecules with both high TS-ensECBS and pharmacophore scores were likely active (Fig. 2b). Taken together, the results suggested that the TS-ensECBS model could serve as a promising primary VS tool.

### Secondary 3D-QSAR Pharmacophore model for complementing the TS-ensECBS model

Despite the high accuracy of the TS-ensECBS model, the in vitro binding assay showed that many of the candidate molecules with a TS-ensECBS score of 0.7 or higher were inactive, suggesting the possibility for further improvement. The training set of TS-ensECBS model contains the randomly chosen negative molecules which are relatively easy to predict with high AUC values. On the other hand, the biochemical binding assay was performed for all positive-like molecules screened by TS-ensECBS. Notably, those selected molecules possibly have several common structural features, which makes the prediction much more difficult with lower AUC values. To complement the TS-ensECBS model and refine the output scores, we constructed a secondary 3D-QSAR model using only the in vitro binding assay data. The structure-based 3D-QSAR model was relatively free from the overfitting problem that occurs in most machine-learning methods, and more importantly, provided the 3D molecular features necessary for optimal target-binding.

**Fig. 4:**
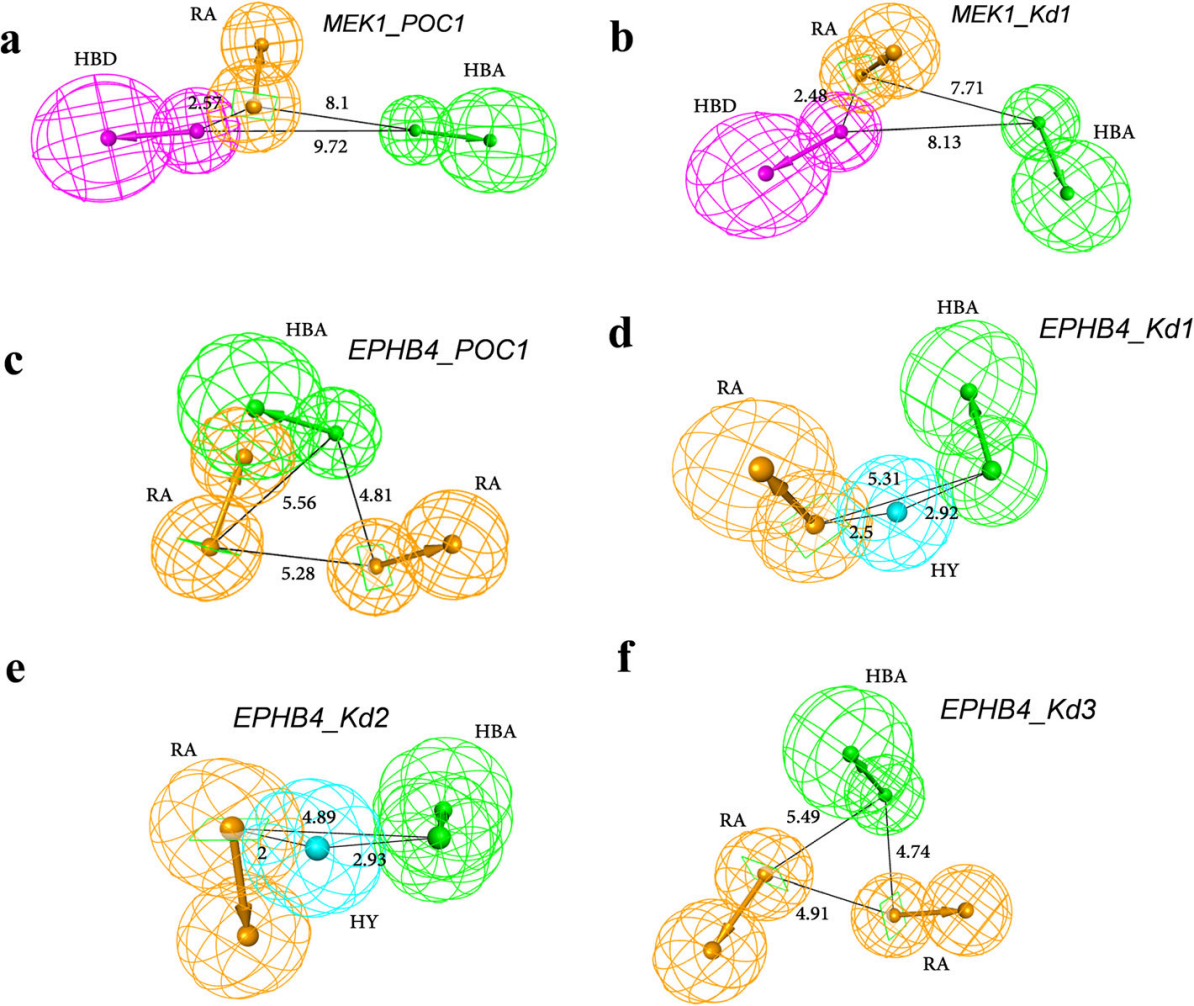
3D-QSAR models generated based on the percentage of control (POC) and binding constant (Kd in nM) values from the biochemical assay. The newly identified lead compounds for MEK1 and EPHB4 were respectively used to create the models. **a** MEK1 model based on POC values. **b** MEK1 model built with binding constant values. **c** EPHB4 model generated via POC values. **d** to **f** EPHB4 models generated using binding constant data. Hydrogen bond acceptor (HBA); hydrogen bond donor (HBD); hydrophobic (HY); and ring aromatic (RA). Every interfeature distance is given as an angstrom

Six reliable 3D-QSAR models built using the competitive binding assay results were finally chosen for MEK1 and EPHB4. The 3D-QSAR pharmacophore models were generated via the Catalyst Hypogen algorithm that used the data (that is the training set, conformational models, pharmacophore features, parameters, and so on) to generate predictive pharmacophores (Fig. 4). All pharmacophores (maximum five features) among the two most active molecules were identified and stored by Hypogen. Pharmacophores that fit the remaining active molecules were retained. Any pharmacophore that matched more than half of the compounds identified as inactive was removed. The highest-scoring unique pharmacophores were exported after optimization. The values of regression analysis by Hypogen, along with the chosen hypotheses, are shown in SI Fig. 4.

**Fig. 5:**
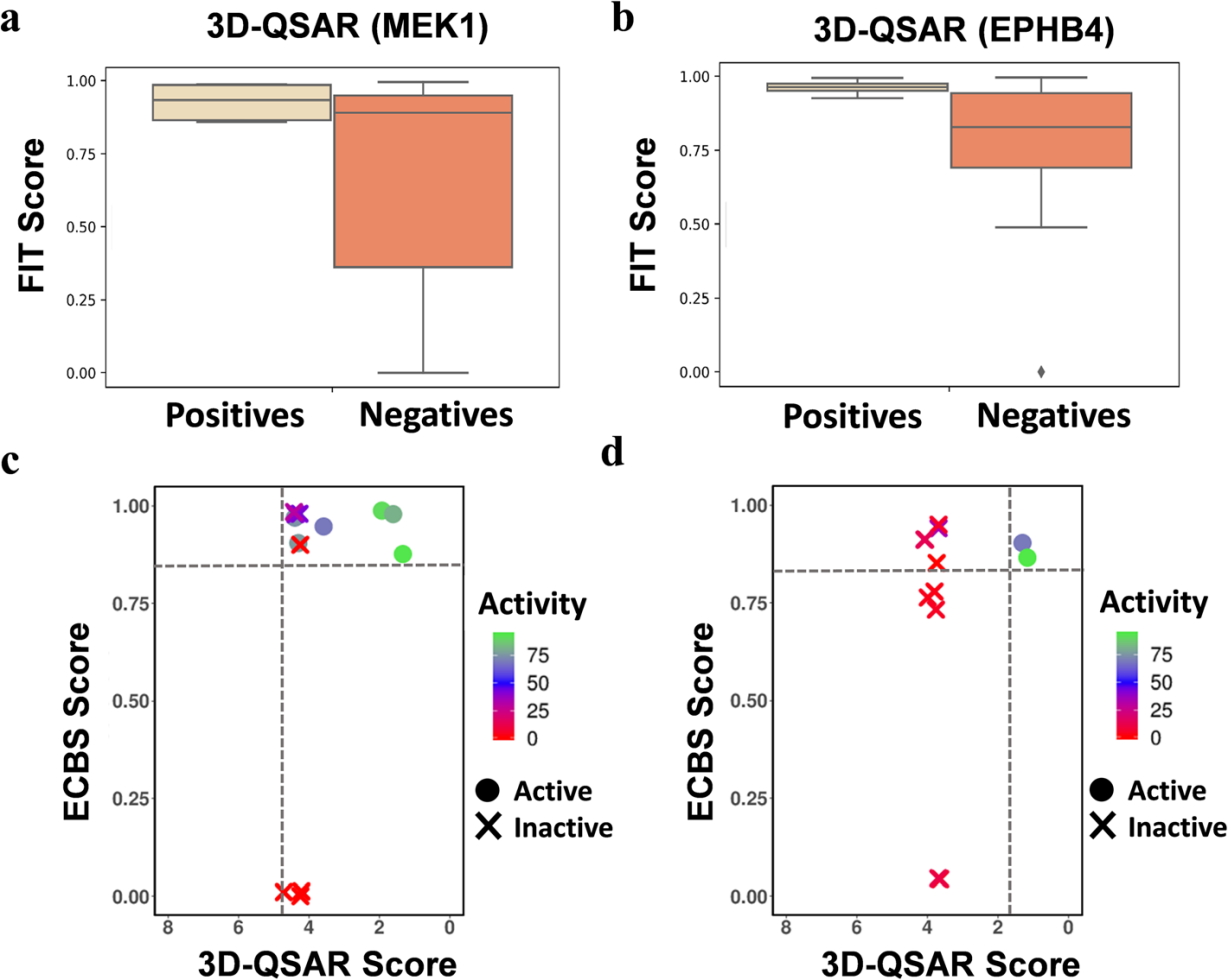
The TS-ensECBS scores and predicted activity values from 3D-QSAR models. **a** and **b** 3D-QSAR pharmacophore models generated based on binding affinity experiments tested with the already reported inhibitors as well as negative molecules that were used earlier to validate the structure-based models. The highest fit value from any of the 3D-QSAR models was selected for each molecule. **c** and **d** Output scores obtained from the TS-ensECBS and 3D-QSAR models for inhibitors confirmed via the competitive binding assay and show activity-based comparisons. The log values of predicted 3D-QSAR activity scores were calculated based on experimental input values (used for 3D-QSAR model generation) for MEK1 and EPHB4. The lowest predicted activity log value from any of the 3D-QSAR models chosen for the target was selected for each molecule

The Fisher randomization option in Discovery Studio (DS) 2018 was used to ensure that the generated hypotheses are acceptable. All the generated models had a statistical significance of 90% or higher, which suggested that the 3D-QSAR models were trustworthy (Fig. 5).

In MEK1 models built based on POC (MEK1_POC1) and Kd (MEK1_Kd1) values, one HBD, one HBA, and one RA were predicted to be common active features among the six active molecules (Fig. 4). The statistical significance for MEK1 models generated based on POC (MEK1_POC1) and Kd (MEK1_Kd1) values were 90 and 95%, respectively (Fig. 4). For the hypothesis using POC values, the EPBH4_POC1 model consisted of two RA features and one HBA feature and its significance was 90% (Fig. 4). In the case of EPHB4 models with Kd values taken as input, the significance was 95% for the three selected hypotheses (Fig. 4). EPHB4_POC1 and EPHB4_Kd3 models had two RA features and one HBA feature while EPHB4_Kd1 and EPHB4_Kd2 models had one HBA, one RA, and one HY feature.

### General applicability of the secondary 3D-QSAR models

Notably, the 3D-QSAR models were not just limited to our experimental data set but were generally applicable to a larger data set without an overfitting problem because the prediction results for the unseen large test set from the kinases still showed a strong discriminative capacity for known binders (Fig. 5a and b). Specifically, the 3D-QSAR models that were built with the MEK1 and EPHB4 inhibitors identified in this study were employed to score the MEK1 and EPHB4 test set molecules used to validate the structure-based methods (Fig. 5a and b). The fit scores of positives in MEK1 and EPHB4 test set molecules for 3D-QSAR models were clearly discriminative against the negatives (Fig. 5a and b). The highest pharmacophore fit score of both positive and negative molecules from any of the selected model was used for calculation. Out of ten MEK1 positive molecules tested, eight had a 0.86 or higher pharmacophore fit score. Similarly, all nine EPHB4 active molecules had a 0.93 or higher pharmacophore fit score. The total number of molecules used to as assess the performance of MEK1 and EPHB4 3DQSAR models were 51 and 49, respectively.

### Combined use of 3D-QSAR and chemical binding similarity

Thanks to the reliability and generality of the 3D-QSAR models, we compared the predicted values of 3D-QSAR models and the chemical binding similarity scores for the MEK1 and EPHB4 inhibitors found in this study to check the possibility of their combined use (Fig. 5c and d). The competitive binding assay results from 13 MEK1 and 12 EPHB4 candidates were used to show the kinase inhibiting properties of molecules. The lowest predicted activity log value from any of the selected 3D-QSAR models for the screened molecules was used for this comparison. The results suggested that the combined use of TS-ensECBS models with the 3D-QSAR model clearly separated the active molecules from the inactive molecules. Accordingly, this strategy for generating a secondary 3D-QSAR model would be useful for further inhibitor screening or in structure-activity relationship studies aiming for new molecules when used along with the ECBS method. As discussed in the individual validation results of both the TS-ensECBS and 3D-QSAR methods in earlier sections, these methods were also individually effective as VS tools against unknown molecules.

### Molecular docking to predict the binding pose of active molecules

**Fig. 6:**
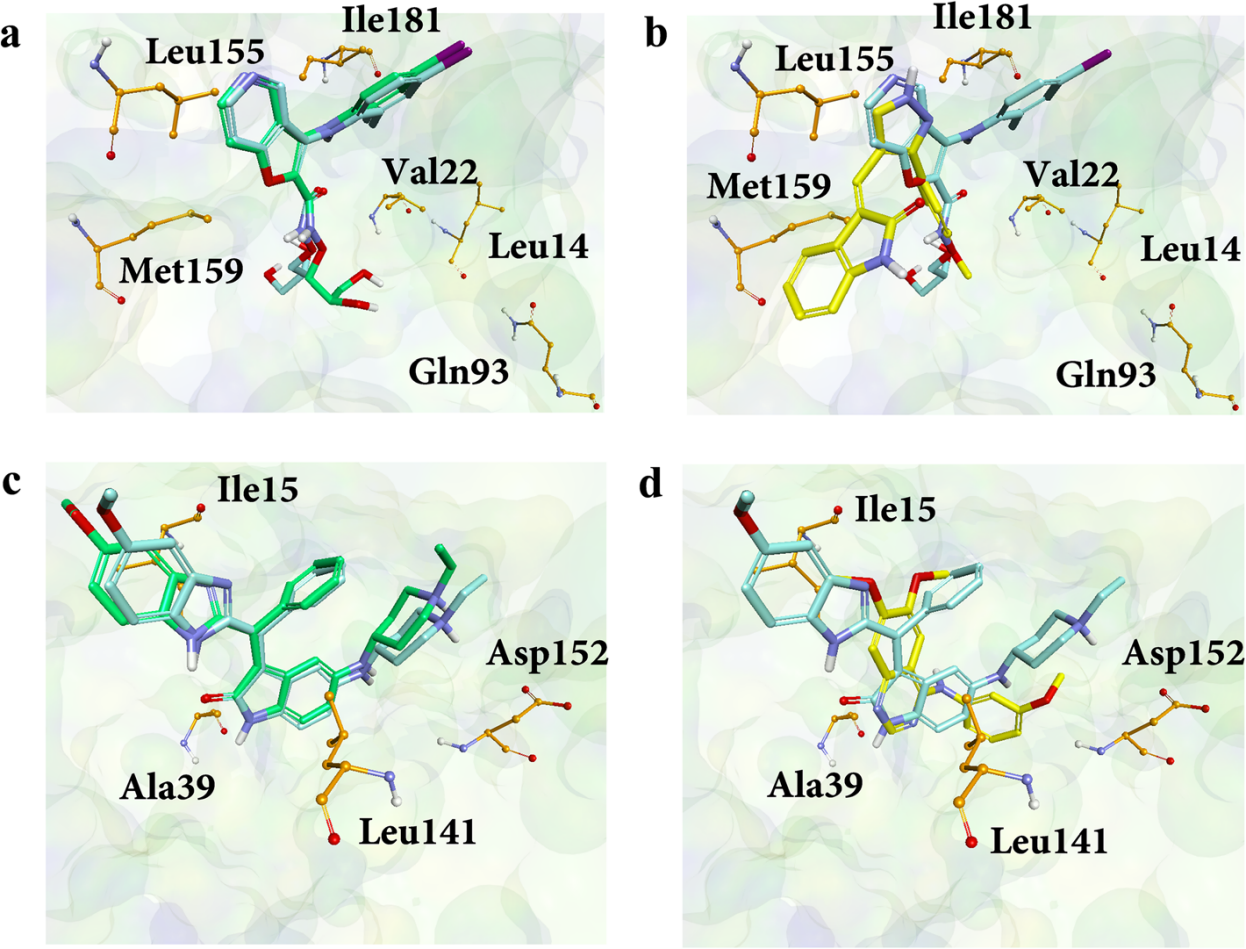
Predicted binding poses of the newly identified inhibitors. The binding modes were predicted using AutoDock Vina. The crystal structures (PDB IDs 3v01 and 4aw5) were used for MEK1 and EPHB4 docking, respectively. Residues that interacted with any of the identified inhibitors are shown as orange ball-and-sticks. The ligands are represented as sticks. Cyan represents the native ligand in the X-ray crystal structure. The ligands shown in green display the predicted binding poses of the native ligands for method validation. The yellow ligands represent the identified inhibitor with the highest binding affinity to its target. **a** The overlay of crystal and the predicted docked pose of MEK1 ligand present in PDB ID 3v01. **b** The ligand in PDB ID 3v01 and the docking result of SEW05801 overlap. **c** The overlay of crystal and the predicted docked pose of the EPBH4 ligand in PDB ID 4aw5. **d** shows the ligand molecule in PDB ID 4aw5 and the docking model of GK03503

Although molecular docking showed poor performance in the VS test, it was useful to predict the binding mode and present the atomic details of protein-ligand interaction when the binding pair was fixed. When the native ligands were docked to the target proteins for a docking pose prediction test, the ligand binding complexes were very close to the corresponding x-ray structures with RMSD values of 1.58 Å and 1.23 Å for MEK1 and EPHB4, respectively. The results suggested the potential use of molecular docking to predict binding modes, even though it could not be used as a primary VS tool based on its binding energy score. The molecules with the highest binding affinity value from the competitive binding assay were chosen to predict the binding models. The binding models of MEK1-SEW05801 and EPHB4-GK03503 complexes are shown in Fig. 6.

In the MEK1 crystal structure, the ligand was involved in bidentate interactions with Ser212 and formed a halogen bond with Val127. In the case of the EPHB4 complex, Asp234 and Met172 formed hydrogen bond interactions with the ligand in addition to several hydrophobic interactions. Binding modes of identified inhibitors in this study suggested that they may not share any conserved interactions with their respective co-crystallized ligands. Their predicted interaction details are discussed below. When molecular docking and 3D-QSAR models for MEK1 were compared, the hydrophobic interactions were disclosed through an RA feature that was similar in all protein-ligand interactions. Specifically, XBX00307 and 18864755 inhibitors were assumed to interact with Val22 and Leu14 of MEK1. The residues Gln93 and Met159 in MEK1 may play a role in binding with XBX00307 and SEW05801, respectively. Interaction with Ile181 may be necessary for GK03503 and GK03409 to bind in the MEK1 cavity. The Leu155 residue in MEK1 may play an essential role in binding to CD09763. For EPHB4 binding, interactions with Ile15, Ala39, Leu141, and Asp152 may be significant for GK03499 and GK03503.

## Discussion

### Integrative approach for virtual screening

In the VS tests based on kinases, the TS-ensECBS models proved effective as a primary screening tool (Fig. 1). The success percentage of the TS-ensECBS models on MEK1 and EPHB4 were 46.2 and 16.7%, respectively, and the TS-ensECBS scores for all experimentally active molecules were high (between 0.86 and 0.98; Table 1). The newly discovered molecules had a low structural similarity to the already reported inhibitors (Table 1) (0.43–0.85 by LiSiCa-2D). However, as previously stated, no hits were confirmed in the competitive-binding assay for WEE1 kinase, which requires further investigation.

The structure-based methods such as 3D-QSAR fitted to the binding assay data may be beneficial to compensate for the limitations of the TS-ensECBS model by rescoring the selected candidates. Although molecular docking was earlier ruled out for primary screening due to its low performance, its binding model prediction for inhibitors could be significant in revealing atomic details for the protein-ligand interactions (Fig. 6).

**Fig. 7:**
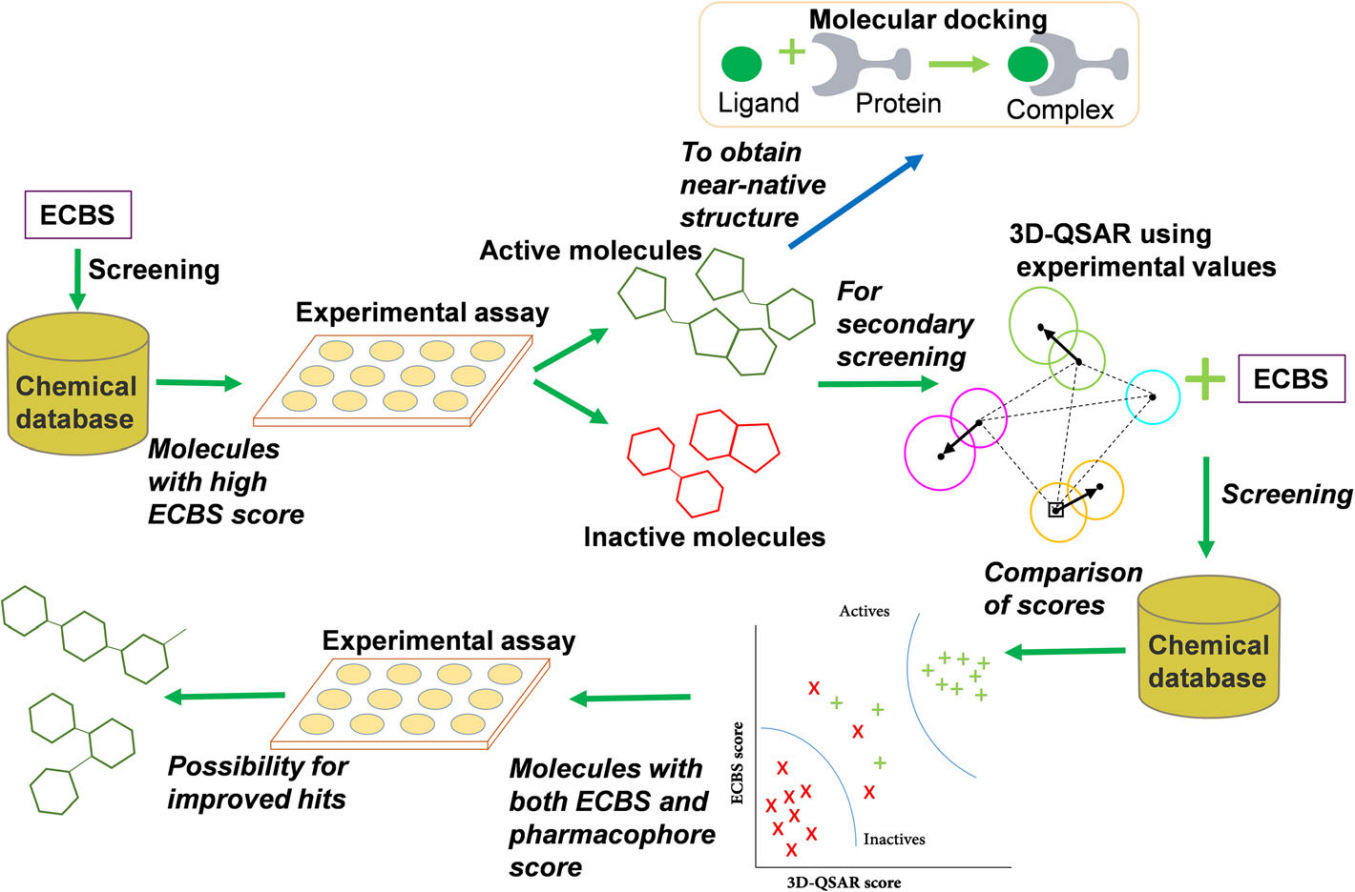
Proposed integrated virtual screening approach. The TS-ensECBS model can be used as a primary initial screening tool owing to its high accuracy. Furthermore, the TS-ensECBS and 3D-QSAR models (built using the obtained experimental data) may be used together for a secondary screening to improve the virtual screening success rate. Molecular docking can be used to predict the protein-ligand binding model for active compounds

Considering the results from the current study, we propose an integrated approach for VS using TS-ensECBS, biochemical assay, 3D-QSAR, and molecular docking models (Fig. 7). The TS-ensECBS model is optimal for an initial screening to perform an in vitro assay. Furthermore, 3D-QSAR models built using experimental data may be used for secondary screening together with TS-ensECBS. Likewise, based on the docking results tested with the known inhibitors of MEK1 and EPHB4, the near-native protein-ligand binding model can be achieved via molecular docking.

### Biological significance of the newly identified MEK1 and EPHB4 inhibitors

Protein kinases have been widely targeted for drugs in the last three decades mainly due to their role in the initiation and development of human cancer [19]. Kinases also play a significant role in several non-cancerous disorders such as immunological and metabolic diseases [19]. In the human genome, 518 kinase genes phosphorylate more than 30% of the proteome, which is essential for signal transduction in various pathways. Thus, kinases pave the way for exploring numerous target-based therapeutic options [19].

Mutations in the genes encoding receptor tyrosine kinases or MEKs lead to deregulation of this signaling pathway, which causes cancer [20]. Thus, inhibitors for receptor tyrosine kinases and MEKs are essential to reduce disease progression. Almost one-third of cancers are proven to have continuous MAPK pathway and MEK1 activation that leads to genetic alteration [21]. MEK inhibitors are also required to clear tumors by immune system reactivation when there are immune evasion and resistance to T cell checkpoint inhibitors [19]. EPHB4 is significant in vascular development but increased EPHB4 expression is found in breast and lung cancers [22, 23]. EPHB4 is also involved in pathological vessel formation such as angiogenesis; hence, molecules to inhibit EPHB4 activity are valuable [24]. Therefore, the newly identified compounds in this study might serve as potential lead compounds for treating various cancers (SI Fig. 5).

## Conclusions

The high success ratio of the TS-ensECBS model in the competitive binding assay (6 out of 13 molecules for MEK1 and 2 out of 12 molecules for EPHB4) suggested that the TS-ensECBS model could be used as a primary VS tool to discover lead compounds for desired targets. Interestingly, several inhibitors identified in this study also have low structural similarity scores to already known inhibitors and thus believed to have novel core structures. Similarly, 3D-QSAR models obtained using the activity data from the in vitro binding assay seemed to complement the TS-ensECBS model by clearly distinguishing the active and inactive compounds. Molecular docking successfully predicted the near-native docking conformations, despite a low VS performance. Hence, the TS-ensECBS model combined with the in vitro binding assay, 3D-QSAR model, and molecular docking should be an effective integrative procedure for VS (Fig. 7). Since the accuracy of proposed combinatorial VS approach needs improvement, involving several other computational methods along with TS-ensECBS may show more valuable results.

## Methods

### Test set generation for kinase targets

First, we shortlisted 395 kinases that were available to test through in vitro binding assays (KINOMEscan, DiscoverX, CA, USA). We selected a subset of the kinases with prebuilt receptor-based pharmacophore models in the PharmaDB database DS2018 [25, 26] that also had protein-ligand complex structures in the PDBbind database [27] for molecular docking simulation. The final test set consisted of 51 kinases; their known active molecules were retrieved from DrugBank [28] and BindingDB [29]. The active molecules in BindingDB were used only when the binding affinity value was below 100 nM by Ki, IC_50_, Kd, or EC_50_ values to exclude any promiscuous binders.

The test set for the TS-ensECBS comparison with ligand-based similarity methods was generated through chemical pairs of the active molecules. That is, the chemical pairs that bound to a common kinase target or evolutionarily related kinase targets were considered positive samples. To generate a negative sample, the fourfold random molecules were sampled from the DrugBank and BindingDB databases for each active molecule. Each of them was paired with the active molecule to generate a negative chemical pair that was unlikely to bind to a common target or evolutionarily related target. The positive and negative chemical pairs were scored by ligand-based similarity methods to compare prediction performance with the TS-ensECBS method.

The test set for the TS-ensECBS method comparison with structure-based methods was separately prepared using the active molecules and randomly sampled (inactive) molecules derived from the previous test set. The individual test molecules (not chemical pairs) were scored using structure-based methods. The TS-ensECBS method was compared to the structure-based methods by assigning the maximum TS-ensECBS score to each test molecule. It should be noted that the TS-ensECBS method outputs a similarity score in terms of binding to the predefined VS target. Redundancy between molecules used for training the TS-ensECBS model and the test set molecules used to validate the methods was checked and removed by comparing InChIKey.

The prediction performance of different VS methods was compared using the AUC values in a PR curve. The PR curve was calculated where Precision = True Positives / (True Positives + False Positives) and Recall = True Positives / (True Positives + False Negatives). The receiver operating characteristic was also calculated where Sensitivity = True Positives / (True Positives + False Negatives) and False Positive Rate = False Positives / (False Positives + True Negatives).

### Virtual screening methods

#### Evolutionary chemical binding similarity

The ECBS model is based on classification similarity-learning [30] where a binary classifier is built for distinguishing evolutionarily related chemical pairs (ERCPs) from unrelated pairs. The chemical pairs are defined as “evolutionarily related” when their targets are identical or have common evolutionary annotation. Diverse evolutionary information defined at multiple levels (e.g., motif, domain, family, and superfamily) is used to annotate binding targets. The classification similarity learning model is then adopted for each evolutionary information to construct a binary classification model distinguish the ERCPs from randomly chosen negative data. The multiple ECBS models built from diverse evolutionary information are integrated by constructing a secondary classifier model. The output prediction scores from each model are combined as training data for the secondary ensemble model (ensECBS) to generate a final output similarity score for a given chemical pair. Among variants of ECBS models, TS-ensECBS is used for VS because the output similarity score is specialized to find chemical pairs binding to a predefined target. In the TS-ensECBS model, the ERCPs are only defined for the targets that are evolutionarily related to a VS target, and the multiple ECBS models based on the target’s evolutionary information are integrated. Accordingly, the ERCPs binding to the VS target are prioritized by the TS-ensECBS model, where the output similarity score estimates the possibility of chemical compounds commonly binding the VS target. The output similarity score ranges from 0 to 1 where the higher similarity represents a higher binding probability. The underlying principles and detailed model construction procedure for the ECBS models can be found in our previous work [17].

TS-ensECBS models were separately created for the 51 kinases including MEK1, EPHB4, and WEE1 and used to score the chemical pairs generated from the input chemical set (all-versus-all pairs). The maximum ECBS score assigned for each molecule was considered as a final target-binding probability.

#### Receptor-based Pharmacophore model

The prebuilt receptor-based pharmacophore models (PharmaDB) incorporated in DS 2018 were used for VS. The pharmacophore features present in PharmaDB were first generated using complex structures by converting them into features such as HBA, HBD, positive ionizable (PI), negative ionizable, HY, and RA. At last, the final 10 models selected using Genetic Function Approximation were considered [31]. More than 117,000 pharmacophore models were included in the PharmaDB database from binding site information in the sc-PDB 2012 database [32].

We used the Ligand Profiler protocol in DS 2018 to screen the PharmaDB models corresponding to the selected kinase test set. For example, the pharmacophore models present in PDB IDs 3v01, 4aw5, and 1x8b were used to select potential inhibitory molecules for MEK1, EPHB4, and WEE1, respectively. The default settings were used in Ligand Profiler except for BEST conformations with a maximum of 255 conformers and flexible fitting type options. Fit values were given as an output score between 0 and 1. The higher the score, the better the fit.

#### Molecular docking

Molecular docking was performed using AutoDock Vina (ADV) [33]. The receptor structures for the selected kinase set were retrieved from the PDBBind database [34]. In ADV, water molecules were removed and the number of rotatable bonds in the ligand was left unmodified. The grid box was set around the bound ligand to perform molecular docking with an exhaustiveness of 20. X, Y, and Z dimension sizes were all 25 Å. X, Y, and Z center coordinates varied for targets according to the bound ligand and target. In cases of flexible docking, side chains of residues around 5 Å from the bound ligand were allowed to repack during the docking simulation. The script “prepare_flexreceptor4.py” provided in MGLTools was used to define the residue flexibility. Molecular docking for MEK1, EPHB4, and WEE1 was performed on the wild type proteins and the native ligands in 3v01 (2.7 Å), 4aw5 (2.33 Å), and 1x8b (1.81 Å), respectively.

#### Chemical structure similarity

Chemical structure similarity was calculated using different methods. The molecular fingerprints, MACCS, and FP4 in the ChemmineOB package [35] were used for similarity calculations. 2D chemical structure similarity was evaluated using the Tanimoto coefficient (i.e. ratio of intersection-bits over union-bits). Moreover, LiSiCA [36], shape-it [37], and LIGSIFT [38] were used with default options to calculate ligand shape similarity. LIGSIFT used Gaussian volumes to calculate the similarity score between molecules irrespective of their size and also calculated the statistical significance for similarity [38]. Shape-it also used Gaussian descriptors for the alignment between reference and query molecules [39]. LiSiCA calculated 2D or 3D chemical similarity through pairwise comparison of chemical features by converting them to molecular graphs [36].

For considering 3D conformational similarity in LiSiCA (−d 3 option) and LIGSIFT, test molecule conformers were generated using the BEST method in DS 2018 with default options (RMSD cut-off: 0.2 Å). The energy-minimized structure obtained using CHARMm force field [40] was compared with each of the 50 low energy conformers and the maximum similarity score was considered a representative similarity score. For LIGSIFT, the ShapeSim score was used to calculate the similarity score.

### Chemical databases for virtual screening

Chemical databases for VS were prepared from marine natural products in MarinLit (22049) [41] and synthetic molecules from Chembridge DIVERSet-CL Library (60000) and Maybridge screening collection (53334) after duplicates were removed. The chemical structures were converted to fingerprint-based features to apply the TS-ensECBS model [35].

### Competitive kinase binding assay

Biochemical experiments were performed through the KINOMEscan service provided by DiscoverX based on a kinase interaction map [42]. Active site (steric or allosteric) binding of a compound was biochemically assessed via the quantity of kinase captured by test and control molecules through a qPCR method. The candidates that bound to kinase impeded the immobilized ligand from binding to the kinase, and thus, the amount of kinase in the bead was diminished. The values of binding between candidates and kinases were given based on a POC, where the lowest percentage of the control (kinase) in solid support confirmed the competition between a test molecule and an immobilized molecule for binding to the kinase. Likewise, the Kd was also obtained via a dose-response curve using the Hill equation [43].

### Construction of the 3D-QSAR pharmacophore model

The POC and Kd values from the in vitro binding assay were used to obtain 3D-QSAR pharmacophore models in DS. The Catalyst Hypogen algorithm [44] in DS predicted the models where binding values helped to provide the “activity property” and “uncertainty property”. The activity value was given same as the experimental values of molecules against the targets. For uncertainty value, if the activity property was 0.001 and the uncertainty property was set to 3.0, the active values were considered to be between 0.001 / 3.0 = 0.00033 and 0.001 × 3.0 = 0.003. So, the uncertainty values for targets were separately chosen based on experimental values of molecules. The “3D QSAR pharmacophore model generation” module in DS 2018 was used with default settings except the activity data were rescaled to 4 orders of magnitude, the energy threshold to 10, and the minimum inter-feature distance to 1.5. The “feature mapping” module was first performed for the active ligands to identify all available features. Further, we chose HBA, HBD, HBY, PI, and RA features for pharmacophore model generation. The 15 molecules retrieved from BindingDB [29] based on varying IC_50_ and Kd values were used for model validation [29]. Fisher validation [45] of 90% was used to assess model quality.

## Supplementary information

**Additional file 1: Figure S1.** The percentage of control (POC) and binding constant values of the final hits. For every inhibitor proved in biochemical assay, its binding constant (Kd in nM) and percentage of control (the lowest percentage of control or kinase in solid support confirms the competition between test molecule and immobilized molecule for binding to kinase) values are shown. **Figure S2.** The dose-response curve used to determine binding constants (Kd). The quantitative measurement by qPCR in y-axis and the corresponding chemical concentration (nM in log10 scale) in x-axis. The “x” mark in the plot represents the data points not used to calculate Kd. **Figure S3.** Diversity of confirmed kinases and binding assay results of WEE1. **a** The diversity of the chosen 3 kinases is shown with the phylogenetic tree of the human kinome. The figure is generated by KinMap website (http://www.kinhub.org/kinmap/). **b** The output scores obtained from TS-ensECBS and PharmaDB screening for the molecules verified via the competitive binding assay for WEE1were shown. **Figure S4.** The experimental and predicted values of 3D-QSAR models. Training set molecules used to generate 3D-QSAR models by Hypogen algorithm with their rescaled competitive binding assay activity values and estimated activity values are shown. The given input activity values from binding assay were rescaled to the range of four orders of magnitude by Hypogen algorithm for better activity prediction. Hence the log values of activity and estimated activity are compared. **Figure S5.** The overview of RAS-directed RAF–MEK–ERK signaling pathway. Initiated by receptor tyrosine kinase RTKs, MEK1 and MEK2 are involved in the pathway for MAPK1/2 activity. This signaling regulates the cell growth where RAS binds to guanosine triphosphate (GTP) and prompts RAF or MEKK to phosphorylate and activate MEKs. Finally, MEKs phosphorylate and trigger ERKs via conserved threonine or tyrosine residues. **Figure S6.** The AUC values in Receiver operating characteristic for the virtual screening methods validated.

## Data Availability

The datasets used and analyzed during the current study are available from the corresponding author (keunwan@kist.re.kr) on reasonable request.

## Abbreviations

ADV: AutoDock Vina
AUC: Area under the curve;
DS: Discovery Studio
ECBS: Evolutionary chemical binding similarity
EPHB4: Ephrin type-B receptor 4
ERCPs: Evolutionarily related chemical pairs
HBA: Hydrogen bond acceptor
HBD: Hydrogen bond donor
HY: Hydrophobic
Kd: Dissociation constant
LiSiCA: Ligand similarity using clique algorithm
MEK1: Mitogen-activated protein kinase kinase 1
PI: Positive ionizable
POC: Percentage of control
PR: Precision and recall
RA: Ring aromatic
TS-ensECBS: Target-specific ensemble evolutionary chemical binding similarity
VS: Virtual screening
WEE1: Wee1-like protein kinase
