# Supplementary material for: Evolutionary chemical binding similarity approach integrated with 3D-QSAR method for effective virtual screening
Source: BMC Bioinformatics. 2020 Jul 14;21:309. doi: 10.1186/s12859-020-03643-x (PMC7362480; doi:10.1186/s12859-020-03643-x)
Supplement: Supplementary file 1 — Additional file 1: Figure S1. The percentage of control (POC) and binding constant values of the final hits. For every inhibitor proved in biochemical assay, its binding constant (Kd in nM) and percentage of control (the lowest percentage of control or kinase in solid support confirms the competition between test molecule and immobilized molecule for binding to kinase) values are shown. Figure S2. The dose-response curve used to determine binding constants (Kd). The quantitative measurement by qPCR in y-axis and the corresponding chemical concentration (nM in log10 scale) in x-axis. The “x” mark in the plot represents the data points not used to calculate Kd. Figure S3. Diversity of confirmed kinases and binding assay results of WEE1. a The diversity of the chosen 3 kinases is shown with the phylogenetic tree of the human kinome. The figure is generated by KinMap website (http://www.kinhub.org/kinmap/). b The output scores obtained from TS-ensECBS and PharmaDB screening for the molecules verified via the competitive binding assay for WEE1were shown. Figure S4. The experimental and predicted values of 3D-QSAR models. Training set molecules used to generate 3D-QSAR models by Hypogen algorithm with their rescaled competitive binding assay activity values and estimated activity values are shown. The given input activity values from binding assay were rescaled to the range of four orders of magnitude by Hypogen algorithm for better activity prediction. Hence the log values of activity and estimated activity are compared. Figure S5. The overview of RAS-directed RAF–MEK–ERK signaling pathway. Initiated by receptor tyrosine kinase RTKs, MEK1 and MEK2 are involved in the pathway for MAPK1/2 activity. This signaling regulates the cell growth where RAS binds to guanosine triphosphate (GTP) and prompts RAF or MEKK to phosphorylate and activate MEKs. Finally, MEKs phosphorylate and trigger ERKs via conserved threonine or tyrosine residues. Figure S6. The AUC values in Receiver o [file 12859_2020_3643_MOESM1_ESM.docx]

**Evolutionary Chemical Binding Similarity Approach Integrated with 3D-QSAR Method for Effective Virtual Screening**

Prasannavenkatesh Durai^1^, Young-Joon Ko^1, 2^, Cheol-Ho Pan^1^, Keunwan Park^1*^

* Correspondence: keunwan@kist.re.kr

^1^Natural Product Informatics Research Center, KIST Gangneung Institute of Natural Products, Gangneung, 25451, Republic of Korea

^2^ Department of Bioinformatics and Life Science, Soongsil University, Seoul 06978, Republic of Korea.


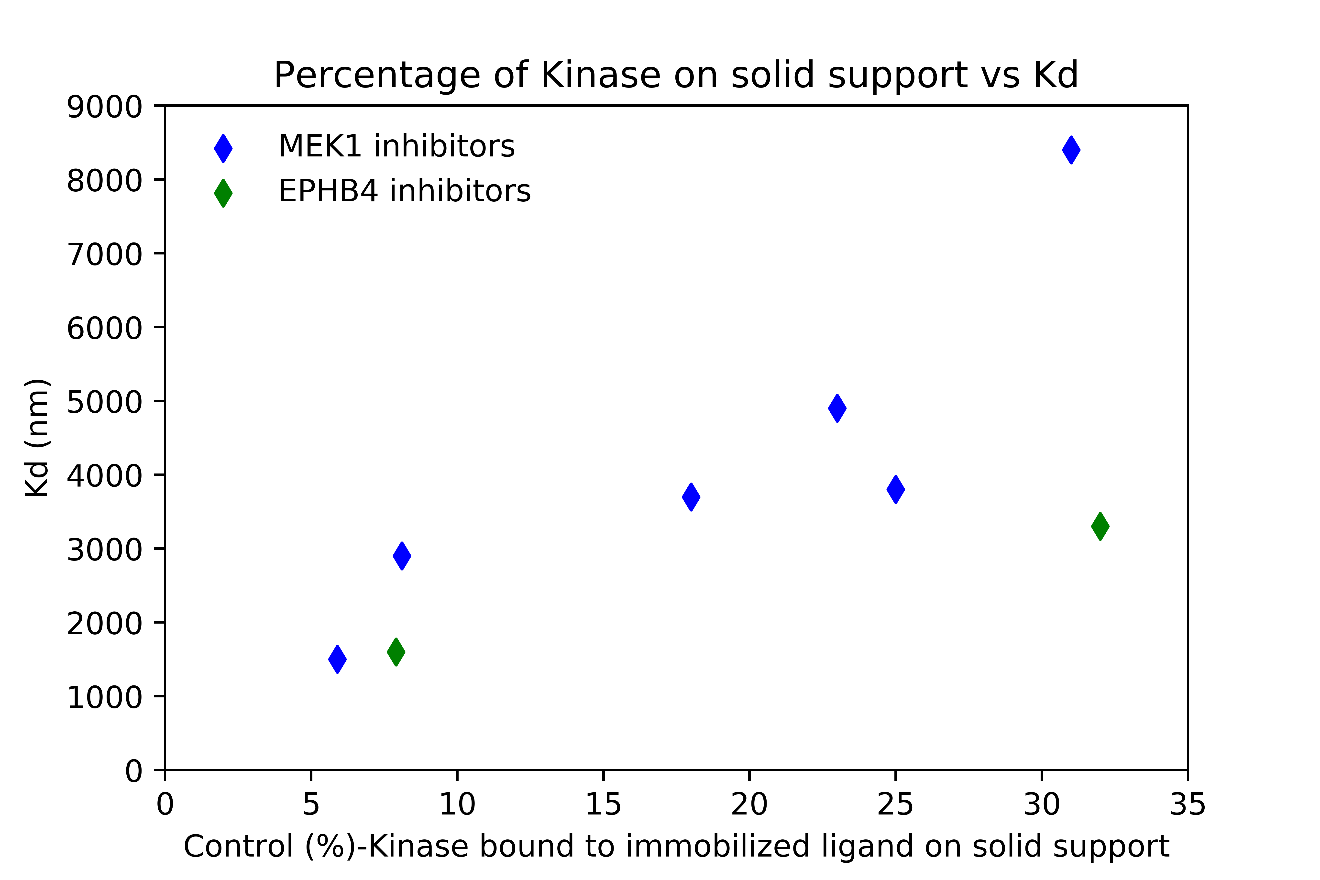


**SI Fig. 1** The percentage of control (POC) and binding constant values of the final hits. For every inhibitor proved in biochemical assay, its binding constant (Kd in nM) and percentage of control (the lowest percentage of control or kinase in solid support confirms the competition between test molecule and immobilized molecule for binding to kinase**)** values are shown.


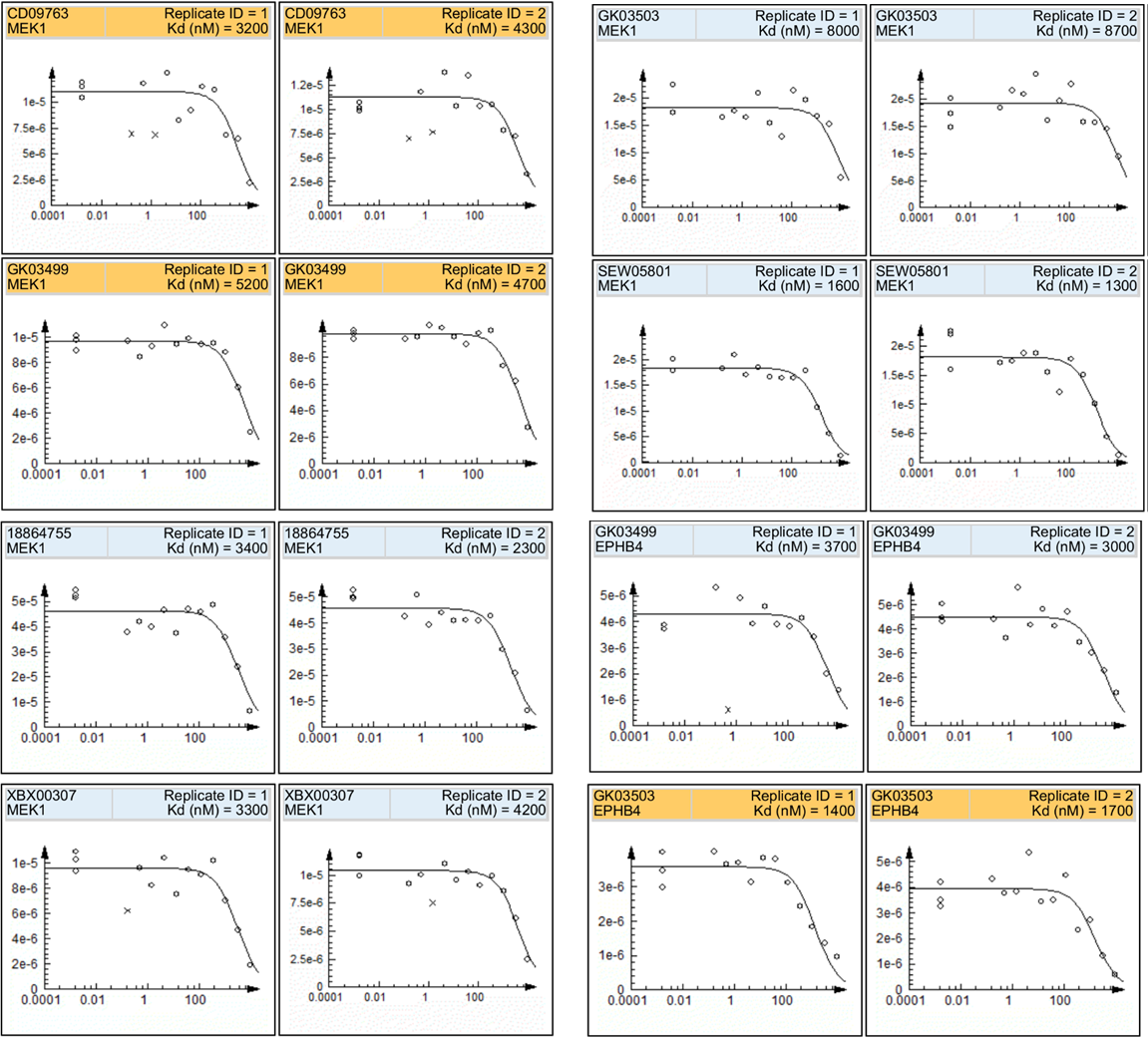


**SI Fig. 2** The dose-response curve used to determine binding constants (Kd). The quantitative measurement by qPCR in y-axis and the corresponding chemical concentration (nM in log10 scale) in x-axis. The “x” mark in the plot represents the data points not used to calculate Kd.


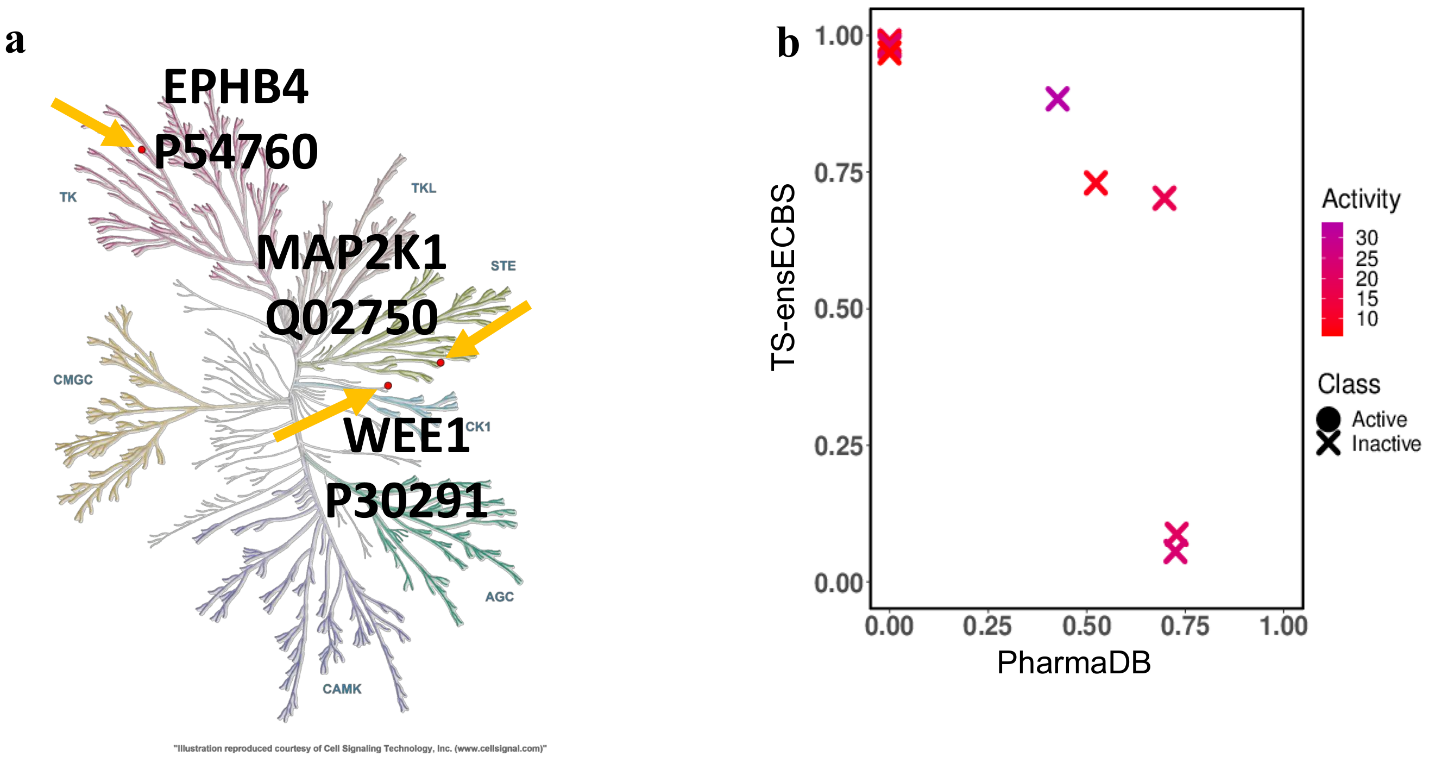


**SI Fig. 3** Diversity of confirmed kinases and binding assay results of WEE1. **a** The diversity of the chosen 3 kinases is shown with the phylogenetic tree of the human kinome. The Fig. is generated by KinMap website (http://www.kinhub.org/kinmap/). **b** The output scores obtained from TS-ensECBS and PharmaDB screening for the molecules verified via the competitive binding assay for WEE1were shown.


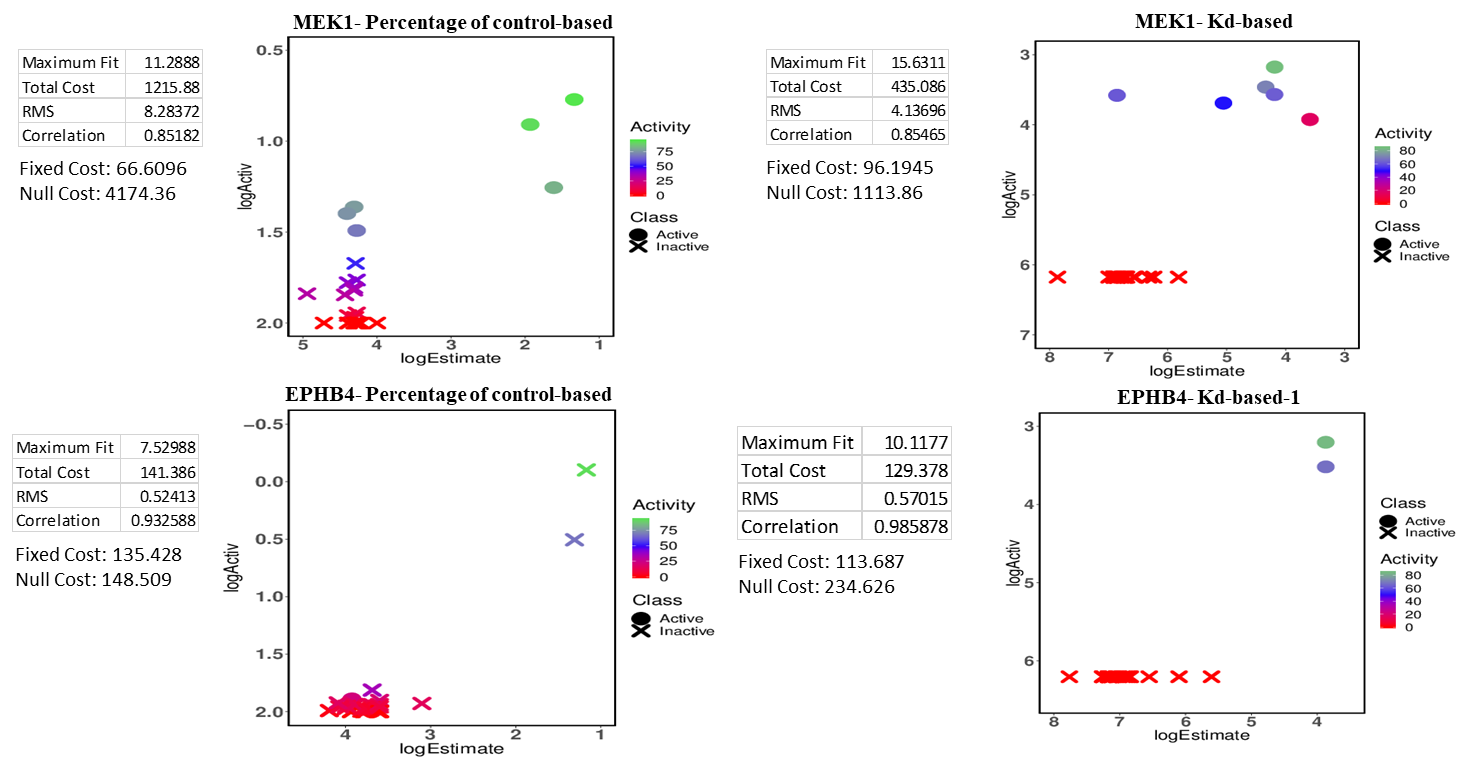


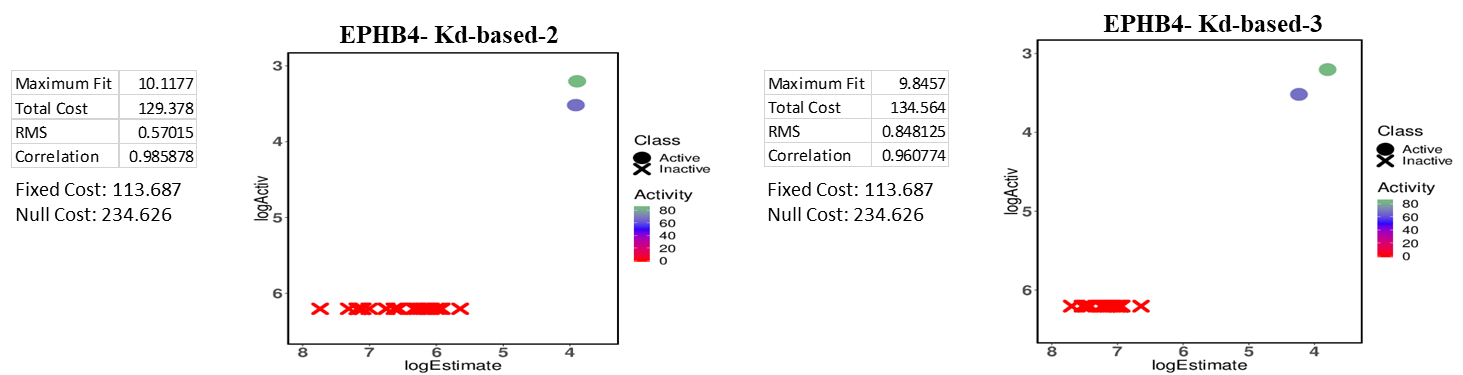


**SI Fig. 4** The experimental and predicted values of 3D-QSAR models. Training set molecules used to generate 3D-QSAR models by Hypogen algorithm with their rescaled competitive binding assay activity values and estimated activity values are shown. The given input activity values from binding assay were rescaled to the range of four orders of magnitude by Hypogen algorithm for better activity prediction. Hence the log values of activity and estimated activity are compared.


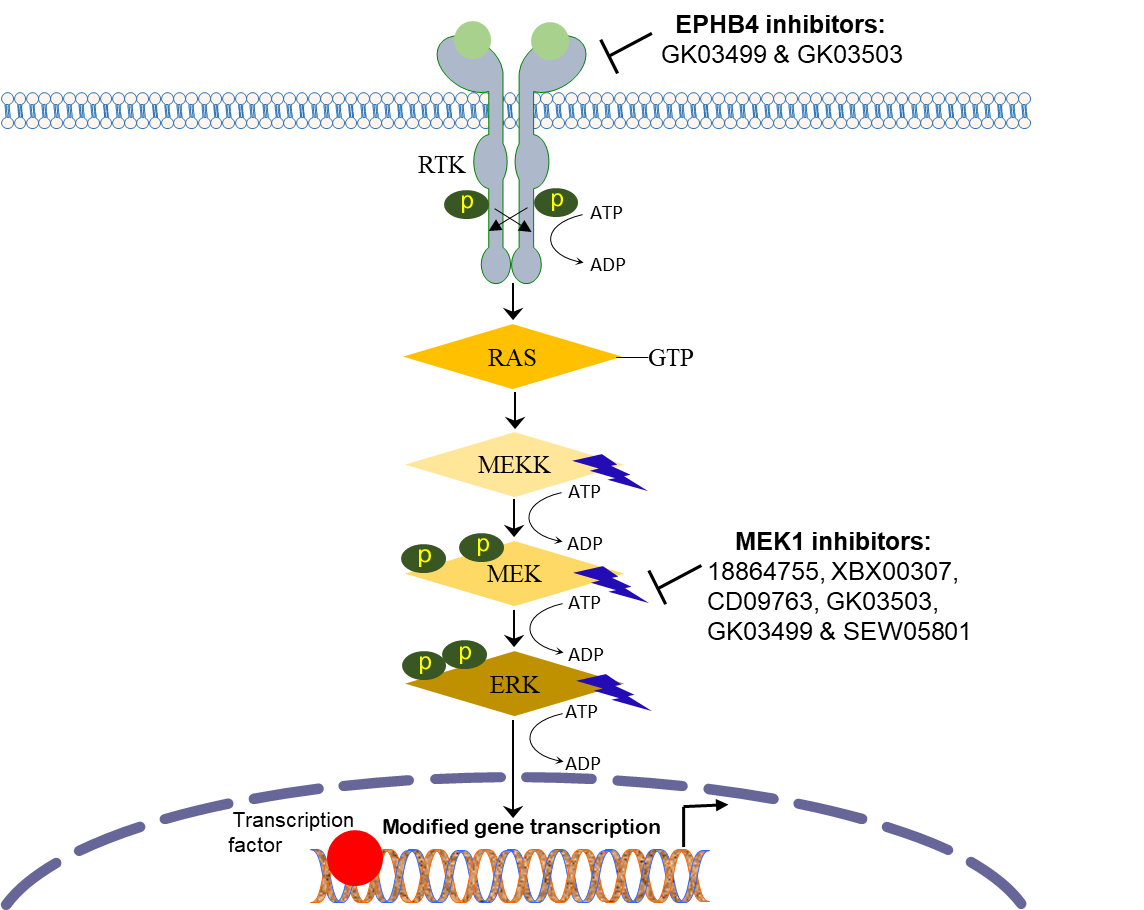


**SI Fig. 5** The overview of RAS-directed RAF–MEK–ERK signaling pathway. Initiated by receptor tyrosine kinase RTKs, MEK1 and MEK2 are involved in the pathway for MAPK1/2 activity. This signaling regulates the cell growth where RAS binds to guanosine triphosphate (GTP) and prompts RAF or MEKK to phosphorylate and activate MEKs. Finally, MEKs phosphorylate and trigger ERKs via conserved threonine or tyrosine residues.


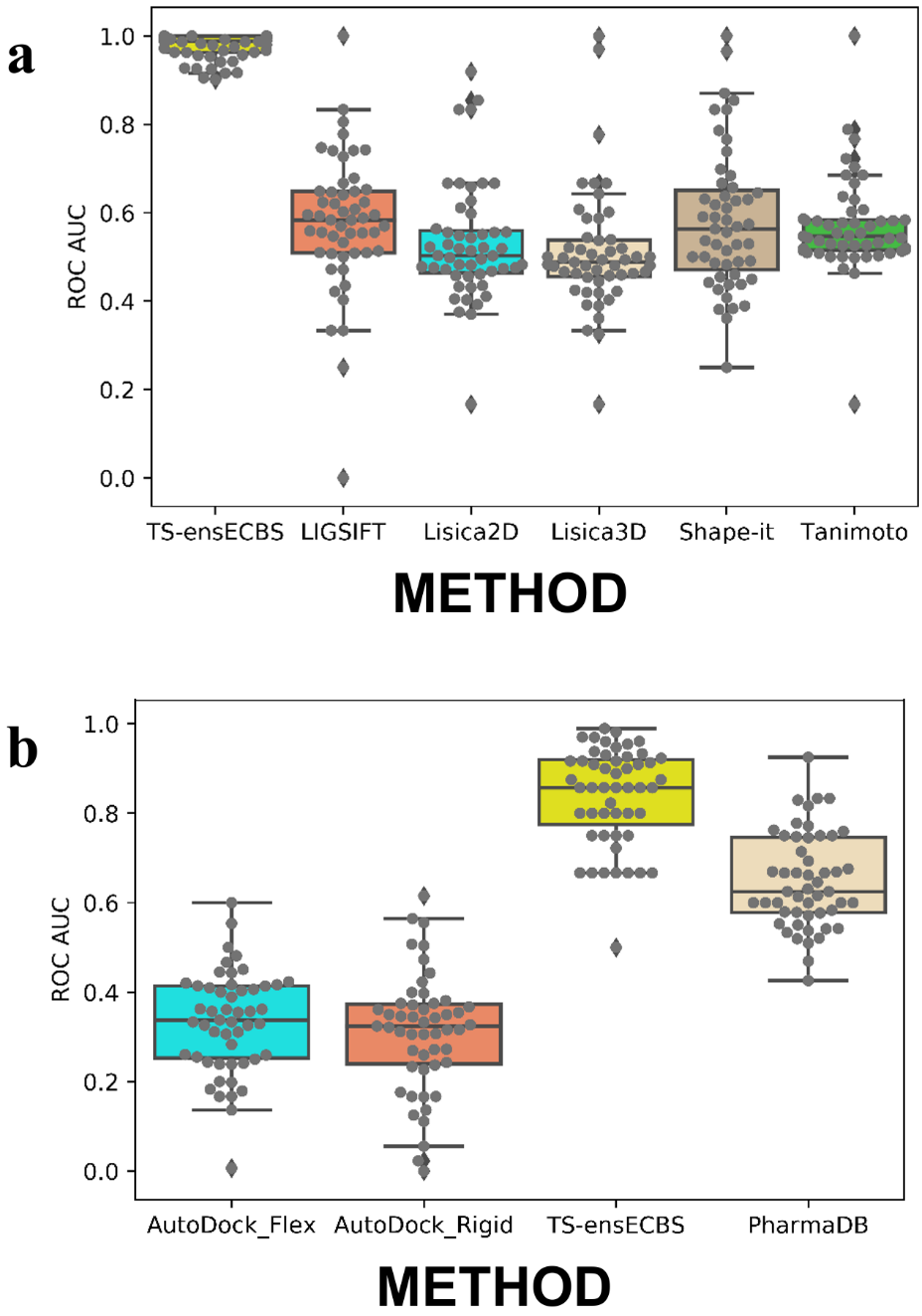


**SI Fig. 6** The AUC values in Receiver operating characteristic for the virtual screening methods validated.
